# 

**Published:** 1887-01

**Authors:** 


					﻿1 1
OLD SERIES
Vol. XXV
NEW SERIES/
Vol. III. —No. II. !
<£>®Us^
1855 O-

■EBM*>r1S

W.F. WESTMOREL AND.«
h.vm.mill£R.m.d.ll.dJR
JAMES A.GRAY. M.D
Published By
JAMES P.HARRISON&CCT
Atlanta, ga. is
SUBSCRIPTION: S2.5O A YEAR.
				

